# An SCF^FBXO28^ E3 Ligase Protects Pancreatic β-Cells from Apoptosis

**DOI:** 10.3390/ijms19040975

**Published:** 2018-03-24

**Authors:** Kanaka Durga Devi Gorrepati, Wei He, Blaz Lupse, Ting Yuan, Kathrin Maedler, Amin Ardestani

**Affiliations:** Centre for Biomolecular Interactions Bremen, University of Bremen, 28359 Bremen, Germany; durga.kpb@googlemail.com (K.D.D.G.); hewei@uni-bremen.de (W.H.); blazpikalupse@gmail.com (B.L.); ting.yuan830@gmail.com (T.Y.)

**Keywords:** pancreatic β-cell, diabetes, human islet, apoptosis, FBXO28, insulin secretion, NeuroD1, E3 ligase

## Abstract

Loss of pancreatic β-cell function and/or mass is a central hallmark of all forms of diabetes but its molecular basis is incompletely understood. β-cell apoptosis contributes to the reduced β-cell mass in diabetes. Therefore, the identification of important signaling molecules that promote β-cell survival in diabetes could lead to a promising therapeutic intervention to block β-cell decline during development and progression of diabetes. In the present study, we identified F-box protein 28 (FBXO28), a substrate-recruiting component of the Skp1-Cul1-F-box (SCF) ligase complex, as a regulator of pancreatic β-cell survival. FBXO28 was down-regulated in β-cells and in isolated human islets under diabetic conditions. Consistently, genetic silencing of FBXO28 impaired β-cell survival, and restoration of FBXO28 protected β-cells from the harmful effects of the diabetic milieu. Although FBXO28 expression positively correlated with β-cell transcription factor *NEUROD1* and FBXO28 depletion also reduced insulin mRNA expression, neither FBXO28 overexpression nor depletion had any significant impact on insulin content, glucose-stimulated insulin secretion (GSIS) or on other genes involved in glucose sensing and metabolism or on important β-cell transcription factors in isolated human islets. Consistently, FBXO28 overexpression did not further alter insulin content and GSIS in freshly isolated islets from patients with type 2 diabetes (T2D). Our data show that FBXO28 improves pancreatic β-cell survival under diabetogenic conditions without affecting insulin secretion, and its restoration may be a novel therapeutic tool to promote β-cell survival in diabetes.

## 1. Introduction

Insulin-producing pancreatic β-cells are key endocrine cells in regulating blood glucose levels and metabolic homeostasis. Progressive loss of β-cell function and/or mass, which is triggered by programmed cell death contributes to the pathogenesis of both type 1 diabetes (T1D) and type 2 diabetes (T2D) [1,2,3] Also, other mechanisms such as β-cell dedifferentiation [4] and defective β-cell proliferation and regeneration [5] are hypothesized as causes for the pancreatic β-cell insufficiency in diabetes. In T1D, β-cells are destroyed by the autoimmune attack of macrophages and T-cells through several deleterious mechanisms such as inappropriately high production of pro-inflammatory cytokines, chemokines, reactive oxygen species (ROS) and other apoptotic stimuli like the perforin/granzyme B and Fas/FasL systems [6]. In T2D, loss of functional β-cell mass is progressive and usually occurs when β-cells are no longer able to provide sufficient amounts of insulin in response to a higher insulin demand under insulin resistance and metabolic pressure. Inappropriately elevated metabolic factors such as glucose, fatty acids and islet amyloid polypeptide (IAPP), together with inflammation induce progressive β-cell dysfunction and death by mechanisms such as endoplasmic reticulum (ER) and/or oxidative stress [7,8,9,10,11]. Consequently, inhibition of β-cell death and dysfunction represents a promising therapeutic approach for the β-cell-directed therapy of diabetes.

The cellular protein abundance and turnover is regulated by the intracellular degradation mechanism called the ubiquitin-proteasome system (UPS). The stability of proteins is controlled by several post-translational modifications, including ubiquitination [12]. By adding small protein ubiquitin to target proteins, they are usually marked for degradation by the proteasome or by the lysosome [13,14]. The intracellular protein degradation program mainly governed by UPS has an essential role in the cell cycle regulation, cell survival, and eradication of misfolded proteins. In pathological conditions, i.e., in major human diseases such as cancer, immunological and neurological disorders [13,14,15] as well as β-cell failure in diabetes [16,17,18,19,20,21,22,23], the UPS machinery is often de-regulated.

The ubiquitin conjugation to a substrate protein is coordinated by a series of enzymatic reactions; an E1 ubiquitin-activating enzyme initiates ubiquitination and transfers ubiquitin to an E2-conjugating enzyme, which then interacts with E3-ubiquitin ligase. E3 carries out the final step, the transfer of ubiquitin to the target protein by the formation of a covalent isopeptide bond between the substrate’s lysine and the carboxyl-terminus of ubiquitin [13,24]. The Skp1-Cul1-F-box (SCF) protein ligase complex is one of the biggest and best described among the E3 ubiquitin ligase family. The SCF complex contains a catalytic core complex comprising of Skp1, Cullin1 and Roc1/Rbx1/Hrt1. F-box proteins are adaptor receptor subunits of this SCF complex and are responsible for recruiting protein substrates through phospho-specific domain interactions [25]. The F-box protein within the E3 ligase complex works as the scaffold connecting the ligase complex with the particular substrates by its F-box domain and substrate binding motif [25]. F-box protein 28 (FBXO28) is an important nuclear F-box protein with incompletely understood cellular functions. Recent research showed that FBXO28 is involved in cell cycle regulation and required for appropriate mitotic progression [26,27]. Loss of FBXO28 results in metaphase to anaphase progression delay, which then leads to several mitotic defects like lagging chromosomes, multipolar spindles and multi-nucleation [27]. A microarray-based analysis identified decreased expression of FBXO28 in human islets isolated from patients with T2D [16]. As the potential action of FBXO28 has never been investigated in diabetes and particularly in the pancreatic β-cell so far, we sought to determine the role of FBXO28 on pancreatic β-cell survival and function under diabetic conditions using clonal β-cells as well as isolated primary human islets from control and diabetic individuals.

## 2. Results and Discussion

### 2.1. Loss of FBXO28 Induces β-Cell Apoptosis

**Figure 1:**
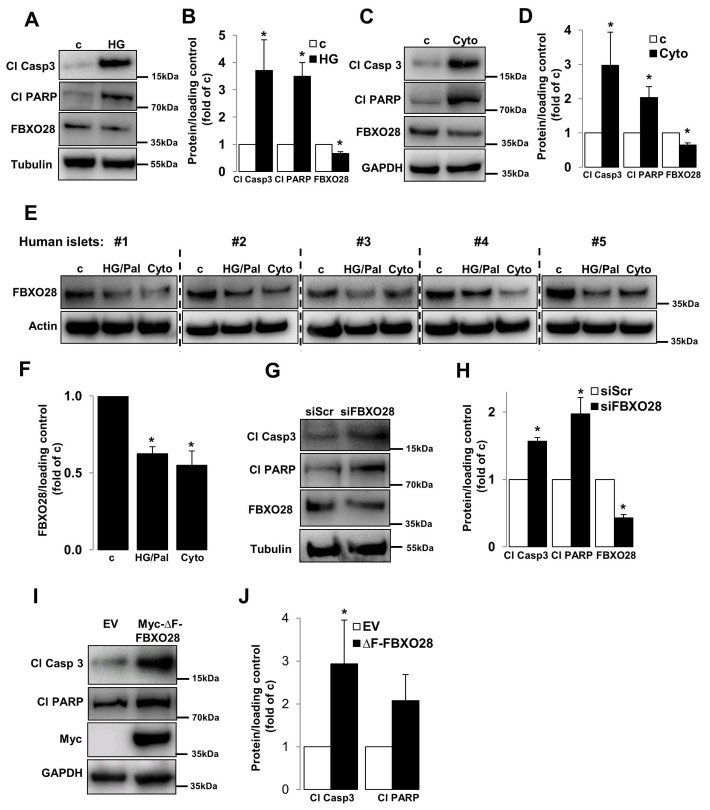
FBXO28 is reduced under diabetic conditions and its knockdown promotes β-cell apoptosis. INS-1E cells or isolated human islets were treated with (**A**,**B**) 22.2 mM glucose (HG), (**E**,**F**) the mixture of 22.2 mM glucose and 0.5 mM palmitate (HG/Pal), or (**C**–**F**) pro-inflammatory cytokines (2 ng/mL recombinant human IL-1β, and 1000 U/mL IFN-γ; cyto) or transfected with either control scrambled siRNA (siScr) or siRNA specific to FBXO28 (siFBXO28, **G**,**H**) or with either control empty vector (EV)- or Myc-conjugated ∆F-FBXO28-overexpressing plasmids (**I**,**J**) for 2 (INS-1E) or 3 (human islets) days. Representative Western blots of cleaved caspase-3 (Cl Casp3), cleaved PARP (Cl PARP) and FBXO28 protein levels (**A**,**C**,**E**,**G**,**I**) and pooled densitometric analyses from at least three independent experiments (INS-1E; **B**,**D**,**H**,**J**) or six human islet preparations (**F**) are shown. GAPDH or Tubulin or Actin was analyzed to ensure equal protein loading. Data show means ± SEM. * *p* < 0.05 compared to control conditions.

Dysregulation of the UPS machinery as well as of its components has been observed in β-cells/islets under diabetic conditions [16,17,18,19,20,21,22,23]. The gene expression of a number of UPS components including several F-box proteins such as FBXO3, FBXO11, FBXW12 and FBXO28 were highly changed in islets isolated from patients with T2D compared to healthy individuals according to previously published microarray-based transcriptome analyses [16]. As impaired β-cell survival is a key pathogenic hallmark of diabetic β-cells [3] and *FBXO28* gene expression level was suggested to be downregulated in T2D islets [16], we sought to investigate the effects of F-box family member, FBXO28, which is expressed in β-cells in the well-established clonal β-cell line INS-1E as well as in isolated human islets (Figure 1) on β-cell survival at basal conditions. FBXO28 protein level was reduced under in vitro treatments commonly used to mimic human diabetes in rodent INS-1E cells (Figure 1A–D), i.e., by elevated glucose concentrations (22.2 mM) and by the cytokine mixture of interleulin-1β (IL-1β), and interferon γ (IFNγ). Consistently, human islets treated with the combination of high glucose and the free fatty acid palmitate as well as pro-inflammatory cytokines show profound down-regulation of FBXO28 protein (Figure 1E,F). In order to understand the physiological impact of such decreased FBXO28 expression under diabetogenic conditions, siRNA was used to knockdown FBXO28 in INS-1E cells. INS-1E cells were transfected with both small interfering RNA (siRNA) against FBXO28 (siFBXO28) or siScr (served as a transfection control). Loss of FBXO28 induced basal β-cell apoptosis, as depicted by increased caspase-3 and PARP cleavage, both well-established markers of apoptosis (Figure 1G,H). In order to test whether the functional F-box domain (which links F-box proteins to the SCF complex via binding to Skp1) in FBXO28 is required for β-cell survival, we transfected the F-box domain-deleted FBXO28 mutant (∆F-FBXO28) into INS-1E cells. Consistent with our results on FBXO28 depletion, overexpression of defective ∆F-FBXO28 induced capase-3 and PARP cleavage in INS-1E cells indicating that functional FBXO28 is essential for maintaining β-cell survival (Figure 1I,J). Altogether, our data demonstrate that FBXO28 expression correlates with β-cell survival and suggest FBXO28 as pro-survival protein in pancreatic β-cells.

### 2.2. Overexpression of FBXO28 Protects β-Cells from Apoptosis

**Figure 2:**
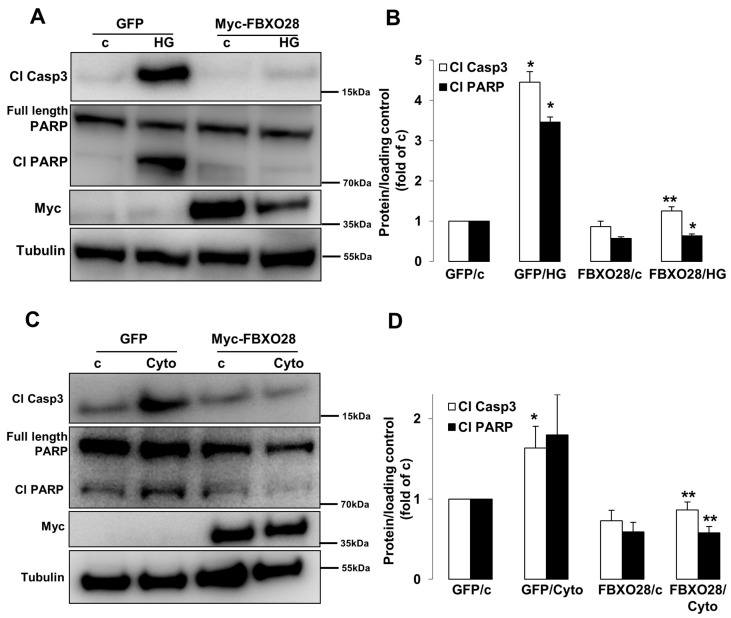
FBXO28 overexpression protects from β-cell apoptosis under diabetic conditions. INS-1E cells were transfected with either control GFP- or FBXO28-overexpressing plasmids and treated with (**A**) 22.2 mM glucose (HG) or (**C**) pro-inflammatory cytokines (2 ng/mL recombinant human IL-1β, and 1000 U/mL IFN-γ; cyto) for 2 days. Representative Western blots of cleaved caspase-3 (Cl Casp3), cleaved PARP (Cl PARP) and Myc protein levels (**A**,**C**) and pooled densitometric analyses from at least three independent experiments (**B**,**D**) are shown. Tubulin was analyzed to ensure equal protein loading. Data show means ± SEM. * *p* < 0.05 compared to GFP transfected control conditions, ** *p* < 0.05 compared to GFP transfected diabetic (HG/Cyto) conditions.

As loss of functional FBXO28 resulted in induction of β-cell apoptosis, we then investigated whether FBXO28 overexpression may restore β-cell survival under diabetogenic conditions. Myc-conjugated FBXO28 was overexpressed by liposome-mediated transfection of INS-1E cells (presented by immunoblotting of Myc; Figure 2) and then cultured under prolonged treatments with high glucose and inflammatory cytokines. Importantly, FBXO28 overexpression improved β-cell survival as indicated by diminished caspase-3 and PARP cleavage under both diabetogenic conditions (Figure 2A–D). Our data show that FBXO28 restoration acts as pro-survival signal to ameliorate the pro-diabetic milieu-induced β-cell apoptosis.

### 2.3. FBXO28 Does Not Regulate β-Cell Function

**Figure 3:**
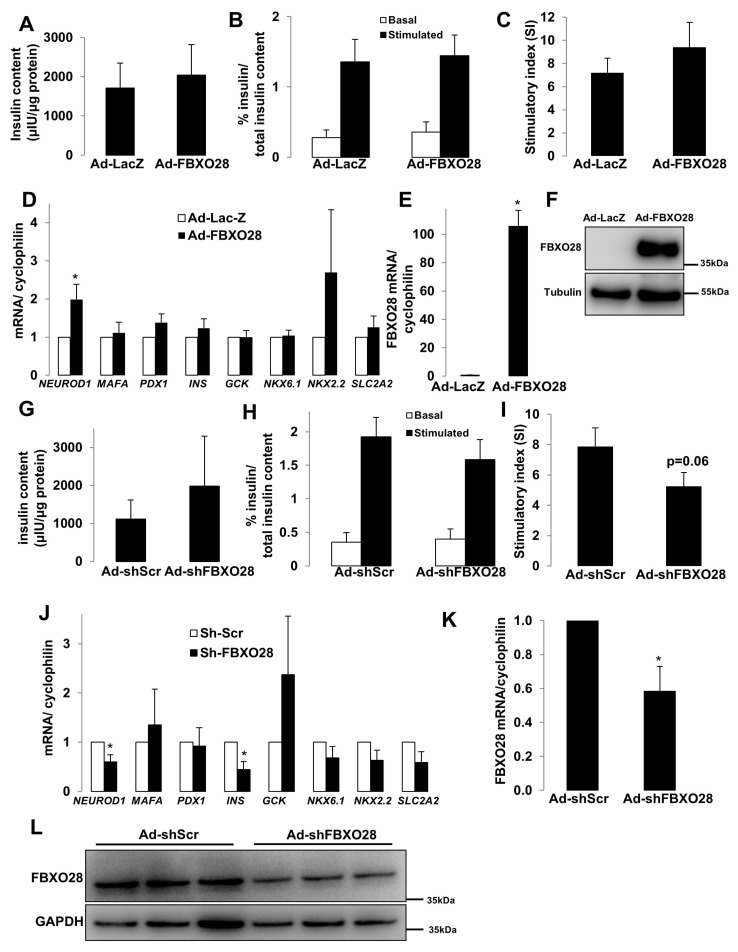
FBXO28 does not regulate β-cell function in human islets. Freshly isolated human islets of nondiabetic organ donors were infected with LacZ control or FBXO28 adenoviruses (**A**–**F**) or with Ad-GFP-shScr control or Ad-GFP-shFBXO28 (**G**–**L**) for 2 days. (**A**,**G**) Insulin content analyzed after GSIS and normalized to whole islet protein. (**B**,**H**) Insulin secretion during 1 h-incubation with 2.8 mM (basal) and 16.7 mM glucose (stimulated), normalized to insulin content. (**C**,**I**) The insulin stimulatory index denotes the ratio of secreted insulin during 1 h-incubation with 16.7 mM and 2.8 mM glucose. (**D**,**J**) RT-PCR for *NEUROD1, MAFA, PDX1, INS, GCK, NKX6.1, NKX2.2* and *SlC2A2* normalized to Cyclophilin. FBXO28 mRNA (**E**,**K**) and protein (**F**,**L**) expression in human islets confirm successful FBXO28 overexpression (**E**,**F**) and downregulation (**K**,**L**). Pooled data are from at least four independent experiments from at least four different human islet donors. Data show means ± SEM. * *p* < 0.05 compared to Ad-LacZ (**D**,**E**) or Ad-shScr (**J**,**K**).

**Figure 4:**
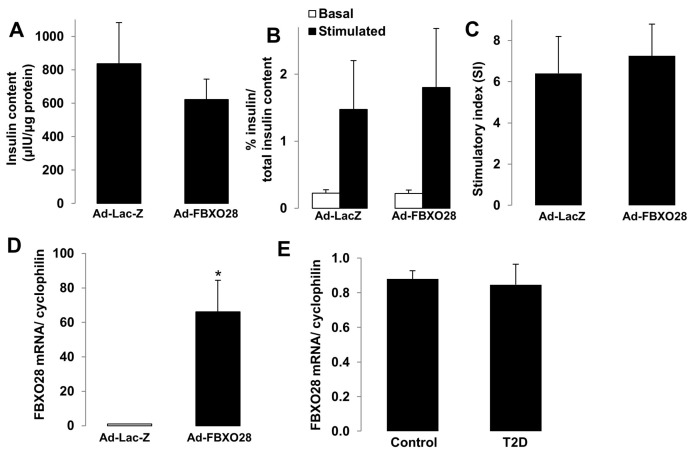
FBXO28 does not improve β-cell function in T2D human islets and *FBXO28* mRNA expression is unchanged in T2D islets. (**A**–**D**) Similar to non-diabetic human islets in Figure 3, freshly isolated human islets from patients with type 2 diabetes (T2D) were infected with LacZ control or FBXO28 adenoviruses for 1 day. (**A**) Insulin content analyzed after GSIS and normalized to whole islet protein; (**B**) Insulin secretion during 1 h-incubation with 2.8 mM (basal) and 16.7 mM glucose (stimulated), normalized to insulin content; (**C**) The insulin stimulatory index denotes the ratio of secreted insulin during 1 h-incubation with 16.7 mM and 2.8 mM glucose; (**D**) *FBXO28* mRNA expression in human T2D islets; (**E**) RT-PCR for *FBXO28* mRNA expression in human islets isolated from nondiabetic (*n* = 24) or individuals with T2D (*n* = 7), normalized to Cyclophilin. (**A**–**D**) Pooled data are from five independent experiments from five different human islet donors with confirmed T2D. Data show means ± SEM. * *p* < 0.05 compared to Ad-LacZ.

The major function of pancreatic β-cells is to control the production of insulin in response to fluctuations in blood glucose levels. To illustrate the potential impact of FBXO28 on the β-cell insulin secretory response and β-cell functional status, we performed glucose-stimulated insulin secretion (GSIS) as well as expression analyses of genes important for β-cell function, identity and maturation. Such essential experiments were performed in human islets isolated from control nondiabetic (Figure 3) and from organ donors with T2D (Figure 4). FBXO28 was overexpressed and silenced in isolated human islets by adenoviral systems. Successful overexpression and short hairpin RNA (shRNA)-mediated depletion of FBXO28 in human islets were assessed using RT-PCR-based analyses of FBXO28 mRNA levels as well as western blotting (Figure 3E,F,K,L) and immunofluorescence for GFP (Figure S1) in infected human islets. In contrast to the FBXO28 effect on β-cell survival, overexpression or knockdown did not significantly alter insulin content, GSIS and insulin stimulatory index in human islets (Figure 3). However, FBXO28 deficiency lowered the insulin stimulatory index by 1.5-fold, compared to infected control islets (Figure 3I), but because of variations among the different human islet isolations, these data did not reach statistical significance (*p* = 0.06). To investigate whether FBXO28 changes modify expression levels of β-cell functional and maturation markers, we analyzed the expression of such genes including key β-cell transcription factors (*PDX1*, *NEUROD1*, *MAFA*, *NKX2.2*, and *NKX6.1*), the hormone insulin (*INS*), as well as important genes for glucose sensing and metabolism (*GCK* and *SLC2A2*) in human islets by RT-PCR (Figure 3D,J). Statistical analysis of pooled quantification data from 4 to 6 different human islet preparations indicated no significant differences for most of the analyzed genes upon FBXO28 knockdown or overexpression, respectively. Only *NEUROD1* highly correlated with FBXO28 expression levels; FBOXO28 overexpression induced *NEUROD1* (Figure 3D; *p* < 0.05), while *NEUROD1* was significantly reduced upon loss of FBXO28 in human islets (Figure 3J; *p* < 0.05), suggesting FBXO28 as previously uncharacterized regulator of *NEUROD1* expression.

Despite the knowledge of NeuroD1 as known regulator of insulin gene expression required for β-cell maturation as well as important factor for β-cell survival [28,29,30], its modest up-regulation upon FBXO28 overexpression did not alter insulin mRNA or protein levels. This is not surprising as insulin gene expression is known to be regulated by a complex transcriptional network involving multiple transcription factors such as PDX1, NeuroD1 and MafA [30,31]. Thus, the up-regulation of one (*NEUROD1*) in the presence of two other unchanged critical transcription factors (*MAFA* and *PDX1*) does not seem to be sufficient to enhance insulin mRNA transcription. Nevertheless, *INS* mRNA levels significantly reduced together with *NEUROD1* upon loss of FBXO28 in human islets according to pooled data from four different human islet preparations (Figure 3J; *p* < 0.05). These data suggest that the mechanism of NeuroD1-dependent insulin gene transcription might be sensitive and operational to the loss of FBXO28, but that an increase of *NEUROD1* alone may not be sufficient to induce insulin production. Further mechanistic experiments are required to disclose such mechanism. Also, despite the tendency of lower expression of some genes including *SlC2A2* and *NKX2.2*, FBXO28 knockdown was not sufficient to significantly change the expression of other genes within the insulin machinery, besides *NEUROD1* and *INS* (Figure 3J), and also did not change insulin secretion itself (Figure 3H). This suggests that compensatory mechanisms for the FBXO28 loss are in place. Altogether, FBXO28 positively regulated *NEUROD1* mRNA expression, but does not have any significant effects on other tested genes, which is consistent with the lack of significant effects on intracellular insulin levels as well as on the insulin secretory response in human islets.

### 2.4. FBXO28 Overexpression Does Not Improve β-Cell Function in T2D Human Islets

The progressive defect in the insulin-secretory response of pancreatic β-cells is a key pathogenic hallmark of β-cell failure in T2D. As FBXO28 protein expression was reduced under diabetogenic conditions (Figure 1) and FBXO28 re-expression could restore β-cell survival (Figure 2), we then hypothesized that FBXO28 overexpression might restore β-cell function in already T2D diabetic islets in the ex vivo setting. Similarly to the experiments in Figure 3 performed in control islets from non-diabetic organ donors, FBXO28 was overexpressed by adenoviral infection of freshly isolated human islets from five different T2D organ donors (Figure 4). Insulin content and GSIS did not change significantly after overexpression of FBXO28 in human T2D islets (Figure 4A–D) indicating that FBXO28 down-regulation is dispensable for the defective insulin secretion in human T2D islets. This is in line with the unaltered insulin secretory function upon changes in FBXO28 expression in human islets from nondiabetic organ donors (Figure 3). Finally, we used our previously collected mRNA from isolated islets from age- and weight matched organ donors with T2D and respective nondiabetic controls for the *FBXO28* mRNA analysis. In contrast to the *FBXO28* reduction observed in islets from patients with T2D, compared to nondiabetic controls from microarray-based transcriptome analyses [16], we could not confirm such *FBXO28* RNA downregulation in T2D islets by classical RT-PCR (Figure 4E). In contrast to these ex vivo data is the downregulation of FBXO28 in β-cells and primary human islets under diabetogenic conditions on the protein level. It is therefore possible, that FBXO28, as substrate recognition partner of the SCF E3 ligase machinery, which mainly acts through its direct-direct protein interaction to recruit its targets, is rather regulated on the protein level, which is confirmed by the diabetogenic conditions in this study.

As a substrate-recruiting domain of the SCF-complex, FBXO28 plays a crucial role in recruiting proteins for degradation or localization in cellular processes such as cell cycle progression and cell proliferation. Recent research demonstrated that FBXO28 expression is regulated during the cell cycle through mechanism including CDK1/2-dependent stabilization [26]. In line with this, FBXO28 is required for proper mitotic progression and cell proliferation, through FBXO28-mediated non-proteolytic ubiquitination of MYC which regulates MYC-dependent transcription [26] and/or direct interaction with topoisomerase IIα, a key enzyme involved in fixing topological constraints of DNA [27]. Consistently, loss of FBXO28 impairs MYC-dependent transcription, hyper-proliferation and neoplastic growth and compromises mitotic progression [26,27]. Whether and to what extent FBXO28 is involved in cell cycle regulation, mitotic progression and proliferation of the hardly-dividing β-cell warrants further mechanistic investigations. By applying FBXO28 loss- and gain-of-function experiments, we show in the present study that: (i) FBXO28 protein levels were reduced in INS-1E β-cell as well as in isolated human islets under diabetic conditions; (ii) loss of FBXO28 as well as overexpression of its defective mutant (∆F-FBXO28) induced basal β-cell apoptosis; and (iii) restoration of FBXO28 was sufficient to confer apoptotic resistance to β-cells under diabetic conditions; (iv) While FBXO28 regulated expression of β-cell transcription factor *NEUROD1* in isolated human islets, it did not alter β-cell function, or expression of several tested β-cell identity and functional genes; (v) Despite its effect on promoting β-cell survival, FBXO28 overexpression did not restore β-cell function in isolated islets from patients with T2D. All this shows that FBXO28 strongly regulated β-cell survival, whereas it did not have any significant independent effect on insulin secretion; neither in non-diabetic nor in T2D primary isolated human islets. This suggests a distinct cellular action of FBXO28 in β-cells. Further studies with the focus on the identification of β-cell specific targets of FBXO28, its underlying mechanism of action as well as thorough investigations of β-cell-specific FBXO28 transgene/knockout mice are under way and will hopefully greatly advance our current understanding of the physiological regulation and function of FBXO28 at the cellular, molecular and organismic level in the control of metabolism.

## 3. Materials and Methods

### 3.1. Islet Isolation, Cell Culture, and Treatment

Human islets were isolated from pancreases of non-diabetic organ donors as well as from individuals with T2D at Lille University and at ProdoLabs and cultured on extracellular matrix (ECM) coated dishes as described formerly [32]. The clonal rodent β-cell line INS-1E was provided by Dr. Claes Wollheim, Geneva and Lund University. Human islets were cultured in complete CMRL-1066 (Invitrogen, Bleiswijk, Netherlands) medium at 5.5 mM glucose and INS-1E cells in complete RPMI-1640 (Invitrogen) medium at 11.1 mM glucose. INS-1E cells and isolated human islets were treated with complex diabetogenic conditions as described previously [3]. Ethical approval for the utilization of islets was granted by the Ethics Committee of the University of Bremen.

### 3.2. Transfections

FBXO28 and ∆F-FBXO28 plasmids [26] (kindly provided by Olle Sangfelt, Karolinska Institute, Stockholm, Sweden) and ON-TARGETplus SMARTpool technology (mix of 100 nM siRNAs directed against rat FBXO28; Dharmacon, Lafayette, CO, USA) were used to overexpress FBXO28/silence FBXO28 in INS-1E cells as described before [3].

### 3.3. Adenoviral Infection

The adenoviruses Ad-h-FBXO28 expressing human FBXO28 and Ad-GFP-U6-hFBXO28-shRNA expressing GFP and human FBXO28 shRNA were obtained from Vector Biolabs (Malvern, PA, USA). Ad-LacZ or Ad-GFP-U6-shRNA were used as respective controls. For transduction, human islets were plated on ECM dishes for 24 h; islets were infected with adenoviruses at multiplicity of infection (MOI) of 100 in FCS-free CMRL-1066 medium. After 4 h incubation, human islets were washed and incubated with fresh complete media. GSIS, RNA and protein extractions were performed 24 or 48 h after infection.

### 3.4. Western Blot Analysis

Western blotting was performed as depicted previously [3]. After the treatment periods, INS-1E cells or human islets were washed twice with ice-cold PBS and lysed with RIPA lysis buffer containing protease and phosphatase Inhibitors (Pierce, Rockford, IL, USA). Protein concentrations were measured by the BCA protein assay (Pierce). Lysates were fractionated by NuPAGE 4–12% Bis-Tris gel (Invitrogen) and electrically transferred onto PVDF membranes. Membranes were blocked in 2.5% non-fat dry milk (Cell Signaling Technology, Danvers, MA, USA) and 2.5% BSA (Sigma, St. Louis, MO, USA) for 1 h at room temperature and incubated overnight at 4 °C with rabbit anti-cleaved caspase-3 (#9664), rabbit anti-PARP (#9532), rabbit anti-cleaved PARP (rat specific #9545), mouse anti-Myc (#2276), rabbit anti-tubulin (#2146), rabbit anti-GAPDH (#2118) and rabbit anti-β-actin (#4967) (all Cell Signaling Technology), and rabbit anti-FBXO28 (#ab154068) (Abcam, Cambridge, UK) followed by horseradish-peroxidase-linked anti-rabbit IgG (Jackson, West Grove, PA, USA). Membranes were developed by a chemiluminescence assay system (Pierce) and evaluated with DocIT^®^LS image acquisition 6.6a (UVP Bio Imaging Systems, Upland, CA, USA).

### 3.5. Glucose-Stimulated Insulin Secretion

Glucose-stimulated insulin secretion (GSIS) was performed in human islets as described previously [3]. Briefly islets were pre-incubated in Krebs-Ringer bicarbonate buffer (KRB) containing 2.8 mM glucose and 0.5% BSA. KRB was then replaced by KRB 2.8 mM glucose for 1 h (basal), followed by an additional 1 h in KRB 16.7 mM glucose (stimulated). Total protein content was extracted with RIPA buffer and protein concentration determined by the BCA assay (Pierce). Insulin levels were measured using human insulin ELISA (ALPCO Diagnostics, Salem, NH, USA). Secreted and intracellular insulin was normalized to total insulin and total protein content.

### 3.6. RNA Extraction and RT-PCR Analysis

RNA was isolated from cultured human islets by utilizing Trizol extraction method (TriFast-PEQLAB Biotechnology, Erlangen, Germany), and cDNA synthesis and quantitative RT-PCR was performed as previously described [3]. For evaluation, we used the Applied Biosystems StepOne Real-Time PCR system (Applied Biosystems, Foster City, CA, USA) with TaqMan(R) Fast Universal PCR Master Mix for TaqMan gene expression assays (Applied Biosystems) for *PDX1* (Hs00236380_m1), *SLC2A2* (Hs01096905_m1), *GCK* (Hs01564555_m1), *INS* (Hs02741908_m1), *NKX2.2* (Hs00159616_m1), *MAFA* (Hs01651425_s1), *NKX6.1* (Hs00232555_m1), *NEUROD1* (Hs01922995_s1), *FBXO28* (Hs00429691_m1), and *PPIA* (Hs99999904_m1).

### 3.7. Statistical Analysis

All values were expressed as means ± SEM and *p* value < 0.05 analyzed by unpaired student *t*-test for comparison of two groups was considered statistically significant.

## Supplementary Materials

Supplementary materials can be found at http://www.mdpi.com/1422-0067/19/4/975/s1.
