# Supplementary material for: An SCFFBXO28 E3 Ligase Protects Pancreatic β-Cells from Apoptosis
Source: Int J Mol Sci. 2018 Mar 24;19(4):975. doi: 10.3390/ijms19040975 (PMC5979299; doi:10.3390/ijms19040975)
Supplement: Supplementary file 1 [file ijms-19-00975-s001.pdf]

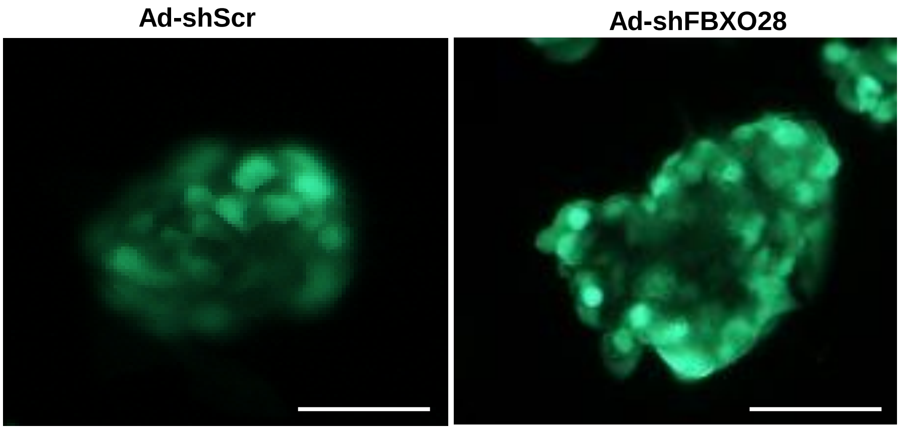

Supplementary Figure 1. Representative immunofluorescence of GFP in infected human islets with Ad-GFP-shScr control or Ad-GFP-shFBXO28. Scale bar represents 50µm.
